# A prospective study of soluble receptor for advanced glycation end products and adipokines in association with pancreatic cancer in postmenopausal women

**DOI:** 10.1002/cam4.1426

**Published:** 2018-03-23

**Authors:** Donna L. White, Ron C. Hoogeveen, Liang Chen, Peter Richardson, Milan Ravishankar, Preksha Shah, Lesley Tinker, Thomas Rohan, Eric A. Whitsel, Hashem B. El‐Serag, Li Jiao

**Affiliations:** ^1^ Department of Medicine Baylor College of Medicine Houston Texas; ^2^ Center for Innovations in Quality, Effectiveness and Safety (IQuESt) Michael E. DeBakey VA Medical Center Houston Texas; ^3^ Texas Medical Center Digestive Disease Center Houston Texas; ^4^ Dan L. Duncan Cancer Center at Baylor College of Medicine Houston Texas; ^5^ Center for Translational Research on Inflammatory Diseases (CTRID) Michael E. DeBakey VA Medical Center Houston Texas; ^6^ Division of Public Health Sciences Fred Hutchinson Cancer Research Center Seattle Washington; ^7^ Albert Einstein College of Medicine Bronx New York; ^8^ Gillings School of Global Public Health University of North Carolina at Chapel Hill Chapel Hill North Carolina

**Keywords:** Biomarker, body weight, CCL2, composite biomarker, pancreatic cancer, prospective, sRAGE

## Abstract

Advanced glycation end products (AGEs) dysregulate adipokines and induce inflammation by binding to their adipocyte receptor (RAGE). Soluble RAGE (sRAGE) prevents AGEs/RAGE signaling. We performed a nested case–control study of the association between sRAGE, adipokines, and incident pancreatic cancer risk in the prospective Women's Health Initiative Study. We individually matched controls (*n* = 802) to cases (*n* = 472) on age, race, and blood draw date. We evaluated serum concentrations of sRAGE, adiponectin, leptin, monocyte chemotactic protein 1 (MCP1), and plasminogen activator inhibitor‐1 (PAI1) using immunoassay. We used conditional logistic regression model to estimate adjusted odds ratios (aORs) and 95% confidence intervals (CIs) for pancreatic cancer over biomarker quartiles (Q1–Q4). We used principal component analysis to create two composite biomarkers and performed a confirmatory factor analysis to examine the association between composite biomarker scores (CBS) and pancreatic cancer risk. Baseline serum sRAGE concentrations were inversely associated with pancreatic cancer risk (aOR_Q4 vs. Q1 _= 0.70, 95% CI: 0.50–0.99). High MCP1 (aOR _Q4 vs. Q1 _= 2.55, 95% CI: 1.41–4.61) and the higher CBS including MCP1, PAI1, and leptin (aOR_Q4 vs. Q1 _= 1.82, 95% CI = 1.04–3.18) were also associated with increased pancreatic cancer risk among women with BMI <25 kg/m^2^ (*P* values for interaction <0.05). We found an inverse association between prediagnostic sRAGE concentrations and risk of incident pancreatic cancer in postmenopausal women. A proinflammatory CBS was associated with increased risk only in women with normal BMI. MCP1 was not modulated by sRAGE.

## Introduction

It is estimated that about 53,670 individuals will be diagnosed with pancreatic cancer in the United States in 2017, and it is the deadliest type of cancer 1. There are several well‐established risk factors for sporadic pancreatic cancer including cigarette smoking, obesity, and a personal history of pancreatitis or type 2 diabetes. These factors are all related to chronic inflammation 2.

Chronic inflammation triggered by the proinflammatory advanced glycation end products (AGEs) and their receptor (RAGE) has been associated with pancreatic carcinogenesis in experimental studies 3, 4, 5, 6. AGEs are glycotoxins generated not only as part of normal metabolism, but also through consumption of foods common in a Western‐style diet (e.g., red meat and high heat processed foods) and via cigarette smoking 7, 8, 9, 10. Endogenous AGE production is increased under conditions of metabolic stress including diabetes and obesity 11. When binding to their cognate receptor, that is, full‐length RAGE, AGEs trigger generation of reactive oxygen species and initiate a downstream proinflammatory signaling cascade including activation of the NF‐κB pathway 12 and contribute to both insulin resistance and chronic inflammation 13. Soluble RAGE (sRAGE) is a decoy receptor which can competitively bind to AGEs and thus prevent the initiation of the proinflammatory RAGE signaling cascade 14. We previously reported a significant, inverse association between baseline sRAGE concentrations and incident pancreatic cancer risk in a prospective cohort of Finnish male smokers 15. However, the generalizability of this finding to other populations is unknown.

In addition to affecting sRAGE levels, AGE accumulation in adipocytes dysregulates production of adipokines, such as adiponectin, leptin, monocyte chemoattractant protein 1 (MCP‐1), and plasminogen activator inhibitor type I (PAI‐1), through binding to RAGE 16, 17, which may affect pancreatic cancer risk. The association between adiponectin and leptin and pancreatic carcinogenesis has been examined in both animal studies and human populations 18, 19, 20, 21, 22, 23, 24. Proinflammatory chemokine MCP1 (aka C‐C Motif Chemokine Ligand 2 or CCL2) is shown to recruit tumor‐associated macrophages for creating an immunosuppressive tumor microenvironment in pancreatic cancer 25. PAI‐1 is a protein encoded by the gene *SERPINE1* and is the principal component of the plasminogen system, which is upregulated in inflammation and cancer 26. However, no epidemiological studies have evaluated the association between MCP1 and PAI1 and risk of pancreatic cancer.

Therefore, we performed a nested case–control study within the prospective Women's Health Initiative Study (WHI) to investigate the association between baseline serum concentrations of sRAGE and adiponectin, leptin, MCP1, and PAI1 and incident pancreatic cancer risk. We hypothesized that sRAGE concentrations would be inverse while proinflammatory adipokines would be positively associated with pancreatic cancer risk, and the concentrations of sRAGE and adipokines were correlated. Furthermore, we evaluated whether the associations were modified by body mass index (BMI). Finally, we used a principal component analysis (PCA) and confirmatory factorial analysis to elucidate the association between sRAGE, adipokines, and pancreatic cancer risk.

## Methods

### Study design and population

Detailed information on the WHI Study including study population, recruitment methods, and measurement protocols has been described previously 27. A total of 161,808 postmenopausal women aged 50–79 were enrolled at 40 clinical centers across the United States between 1993 and 1998. A total of 68,132 women were enrolled in four overlapping WHI Clinical Trials (WHI‐CTs) on calcium/vitamin D, a low‐fat dietary modification (DM) intervention, and hormone replacement trial. A total of 93,676 women who were not enrolled in the clinical trials formed an Observational Study (WHI‐OS). Our study participants were drawn from the WHI‐OS and WHI‐CT except for the active arm of the dietary modification trial because we found dietary modification potentially reduced pancreatic cancer incidence 27.

### Ascertainment of pancreatic cancer case

The WHI maintains ongoing follow‐up for health outcomes; for this analysis, we used data through December 2013. Cases of incident pancreatic cancer were ascertained from annual self‐administered questionnaires. The diagnosis was further verified via a centralized review of medical records including clinical notes, pathology, operative and radiology reports, and tumor registry abstracts and eventually adjudicated according to the International Classification of Diseases for Oncology site codes C25.0–C25.4, C25.7–C25.9 28.

### Ascertainment of matched controls

**Figure 1:**
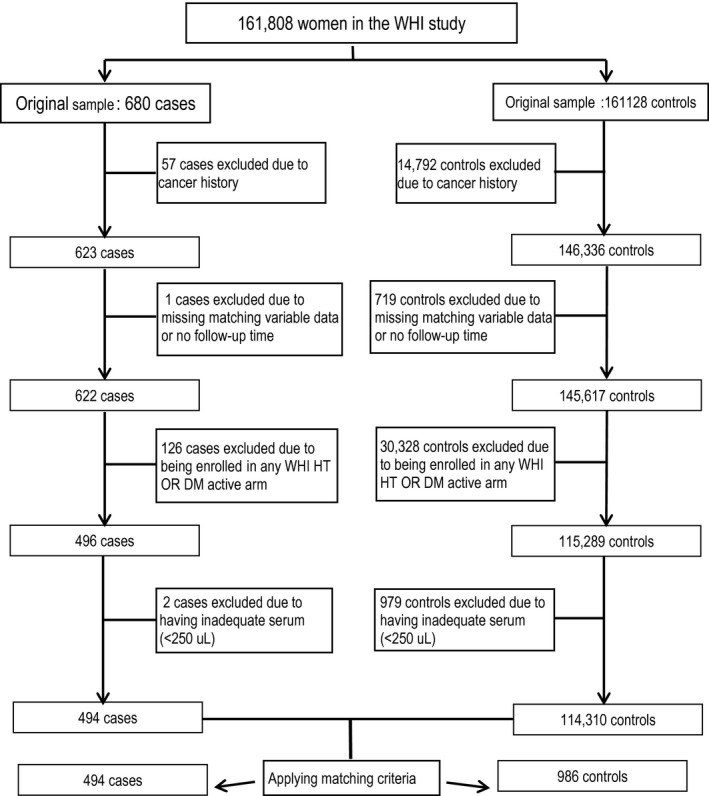
Flowchart for sample selection for the current analysis.

We identified 680 incident pancreatic cancer cases and 161,128 controls who had no cancer history from the original cohort (Fig. 1). To increase the internal validity of our analyses, we excluded women who reported having cancer (other than nonmelanoma skin cancer) (*n* = 57 cases and *n* = 14,792 controls) prior to baseline, who had missing matching variable data or follow‐up time (*n* = 1 cases and *n* = 719 controls), who were in DM active intervention arm (*n* = 126 cases and *n* = 30,328 controls), or who had inadequate serum (*n* = 2 cases and *n* = 979 controls). Thus, we identified 494 cases and 114,310 potential controls (Fig. 1). We randomly selected two cancer‐free controls from the risk set to be individually matched to each case. Matching criteria were age at baseline (±3 years), ethnicity, baseline blood draw date (±6 months), OS enrollment (yes/no), Hormone Therapy (HT) trial arm (yes/no), calcium and vitamin D trial arm (yes/no), hysterectomy at baseline (yes/no), and study center.

The initial study population selected for this investigation included 1480 women (494 cases and 986 matched controls). However, in the main analysis, we excluded 186 controls and 22 orphaned cases (without matching controls) because they developed breast, colorectal, or lung cancers after the index diagnosis date of the matched pancreatic cancer case. Thus, our final analytic cohort included 142 and 330 cases matched 1:1 and 1:2 to controls, respectively, yielding a total of 472 cases and 802 matched controls.

### WHI data collection and exposure assessment at baseline

Detailed information including sociodemographic characteristics, personal medical history, reproductive history, and lifestyle including smoking and alcohol use was obtained at baseline using interviewer‐administered structured questionnaires 29. Participants also completed a validated food frequency questionnaire about their food consumption in the last 3 months 29. Anthropometric measurements including weight, height, and waist circumference were obtained. Participants brought in bottles for medications and supplements they used to enable determination of medication use.

### Blood collection and measurement of circulating biomarkers

Blood samples obtained at baseline following an overnight fast of at least 12 h were stored at −70°C. Because serum samples have been used to test biomarkers previously, all samples have gone through the same freeze‐thaw cycles (less than two cycles).

We used human sRAGE Quantikine ELISA kit (R&D System Inc., Minneapolis, MN) to measure sRAGE concentrations. We used the R&D Human Obesity MultiAnalyte Profiling Kit (R&D System Inc.) to concomitantly measure concentrations of adiponectin, leptin, MCP1, and PAI1. All kits had the same lot number. All the biomarkers were measured at Baylor College of Medicine. Randomly ordered case and matched control samples were run side by side in the same batch (96‐well plate). A 10% quality control (QC) sample provided by the WHI clinical coordinating center was also randomly placed on each plate. Laboratory personnel who performed the ELISA assay were blinded to QC samples and case–control status. All samples were assayed in duplicate. The QC samples showed that all inter‐ and intra‐assay CVs varied from 2% to 5% for all biomarkers indicating excellent laboratory performance.

The study protocol was approved by Institutional Review Board (IRB) of the WHI and by the combined IRB of Baylor College of Medicine and the Michael E. DeBakey VA Medical Center in Houston, TX.

### Statistical analysis

We compared the distribution of sociodemographic factors, anthropometric characteristics (BMI, waist‐to‐hip ratio (WHR)), medical history (personal history of diabetes, pancreatitis, family history of cancer, use of NSAIDs), lifestyle factors (smoking status, >1 drink alcohol per day), food consumption (e.g., energy‐adjusted daily intake of carbohydrate and total and saturated fat, daily servings of red meat, fruits, vegetables), medication use (beta‐blocker, statin, and angiotensin‐converting‐enzyme inhibitor (ACEi)), and baseline concentrations of circulating biomarkers (sRAGE, adiponectin, leptin, MCP1, and PAI1) in pancreatic cancer cases and matched controls using paired t‐tests or Wilcoxon signed‐rank tests for normal or non‐normal distributed continuous variables, respectively, and McNemar's or Cochran's Q test for categorical variables for two or more than two categories, respectively.

We used the distribution of each circulating biomarker in controls to define quartile (Q) thresholds. We investigated the association between our primary biomarker of interest, sRAGE, and all other biomarkers (adiponectin, leptin, MCP1, and PAI1) as well as the ratio of adiponectin to leptin (adiponectin/leptin) and risk of incident pancreatic cancer using conditional logistic regression models. We performed staged multivariable analysis with the minimal multivariable model adjusting for history of type 2 diabetes and smoking (current/former vs. never) as they are established risk factors for pancreatic cancer. BMI (<25, 25–29, 30+ kg/m^2^) was further adjusted in the model to evaluate its mediate effect on sRAGE and risk of pancreatic cancer. All other variables evaluated in Table 1 did not change risk estimates by more than 5% and thus were not included in final multivariable models. Risk estimates were reported as odds ratio (OR) with associated 95% confidence interval (CI).

**Table 1:** Baseline characteristics of pancreatic cancer cases and cancer‐free controls in the WHI Study, 1993–2013

Characteristics (mean (SD) or %)	Cases (*n* = 472)	Controls (*n* = 802)	*P* value
Age at randomization (year)	65.4 (7.1)	65.3 (7.1)	0.91
Race, % non‐Hispanic White	409 (86.7)	689 (85.9)	0.71
Education, % college or post graduate	331 (70.1)	542 (67.6)	0.64
BMI (kg/m^2^)	27.8 (5.7)	27.1 (5.6)	0.03
BMI (kg/m^2^)			
BMI <25	168 (35.6)	301 (37.5)	0.11
BMI 25–30	173 (36.6)	320 (39.9)	
BMI ≥30	131 (27.8)	181 (25.6)	
Waist‐to‐hip ratio (WHR)	0.82 (0.08)	0.81 (0.08)	0.22
WHR			
WHR <0.8	226 (47.9)	384 (52.1)	1.00
WHR ≥0.8	246 (47.9)	418 (52.1)	
Smoking status			
Never smokers, %	237 (50.2)	428 (53.4)	0.35
Former smokers, %	195 (41.3)	315 (39.3)	
Current smokers, %	36 (7.6)	48 (6.0)	
Missing, %	4 (0.9)	11 (1.3)	
Physical activity (MET‐hour/week)	12.4 (12.8)	13.4 (14.2)	0.22
NSAIDs use, % yes	102 (21.6)	166 (20.7)	0.70
Treated type 2 diabetes, % yes	30 (6.4)	33 (4.1)	0.08
Pancreatitis, % yes	3 (0.6)	4 (0.5)	0.75
2nd‐degree family history of known cancers, % yes	312 (66.1)	527 (65.7)	0.89
Alcohol, % >1 drink/day	70 (14.8)	98 (12.2)	0.18
Total energy intake, Kcal	1586 (688)	1606 (692)	0.61
Protein, g/1000 Kcal	42.4 (8.2)	41.8 (7.6)	0.24
Total carbohydrate, g/1000 Kcal	125 (22.9)	128 (23.2)	0.04
Available carbohydrate, g/1000 Kcal	115 (20.8)	117 (20.9)	0.04
Total sugar, g/1000 Kcal	61.4 (18.7)	63.0 (19.0)	0.15
Total fat, g/1000 Kcal	35.8 (9.4)	35.5 (9.4)	0.52
Saturated fat, g/1000 Kcal	12.0 (3.7)	11.7 (3.6)	0.13
Red meat (servings/day)	0.68 (0.6)	0.66 (0.5)	0.52
Total vegetables (serving/day)	1.55 (0.9)	1.54 (1.0)	0.78
Total fruits (serving/day)	1.30 (0.8)	1.33 (0.9)	0.41
HRT trial assignment (active) (*N*, %)	32 (6.8)	57 (7.1)	0.97
Calcium/vitamin D intervention assignment (active) (*N*, %)	45 (9.5)	92 (11.5)	0.55
sRAGE (pg/mL)	1366 (588)	1436 (616)	0.04
Adiponectin (*μ*g/mL)	12.5 (8.3)	12.6 (6.9)	0.80
Leptin (ng/mL)	30.2 (25.4)	31.4 (28.3)	0.88
MCP1 (pg/mL)	239.0 (124)	234.4 (144)	0.57
PAI1 (ng/mL)	78.8 (24.5)	76.7 (26.9)	0.17

BMI, body mass index; HRT, hormone replacement therapy; MET, metabolic equivalent of task; NSAID, nonsteroidal anti‐inflammatory drugs; WHR, waist–hip ratio.

We performed stratified analyses to further assess for potential effect modification by two variables *specified* a priori: BMI (<25 vs. ≥25 kg/m^2^) and follow‐up time (<10 years vs. ≥10 years). The score test was used to assess for trend across increasing quartiles by treating the quartile levels as a continuous variable with significance of interaction terms evaluated by a Wald test. In the stratified analysis, the case–control match was broken. The primary matching factors age and race/ethnicity were adjusted for in the unconditional logistic regression models. Sensitivity analyses were performed by excluding study participants who had <1 year of follow‐up (450 cases and 801 controls retained) or including controls who later developed cancers of breast, colon‐rectum, and lung and their matched pancreatic cancer cases (494 cases and 986 controls).

We used log transformations to normalize the distributions of sRAGE, adiponectin, leptin, MCP1, and PAI1 and calculated the Pearson correlation coefficients and associated *P* values for all the biomarkers with BMI. We further conducted PCA that showed a strong two‐dimensional structure. Oblique rotations were applied to the two principal components to ascertain a structure that would eliminate low correlations between these two factors. This structure was then used in specifying a confirmatory factor analytic (CFA) model (a multiple regression of the biomarkers on the two factors as latent variables). The scores from the two factors obtained from these CFA regressions were then used as composite biomarker scores to serve as surrogates for the original biomarkers in multivariate conditional logistic regression analysis assessing pancreatic cancer risk.

All statistical tests were two‐sided with *P* values considered significant at *P *<* *0.05 because sRAGE was the main biomarker of interest, and with all analyses performed using SAS version 13 (SAS Institute, Cary, NC).

## Results

Table 1 shows that the distribution of most potential confounding variables was comparable between cases and controls. However, the cases had higher mean BMI than controls and had lower consumption of total and available carbohydrate than controls. Mean baseline serum sRAGE concentrations were significantly lower in cases than in controls (*P *=* *0.04).

Table 2 shows postmenopausal women with baseline sRAGE concentrations in the highest quartile had significantly reduced risk of developing incident pancreatic cancer (aOR_Q4 vs. Q1 _= 0.70, 95% CI: 0.50–0.99; *P* trend = 0.03). The association was slightly attenuated with further inclusion of BMI in the model. There was a suggestive inverse association with adiponectin (aOR _Q4 vs Q1 _= 0.74, 95% CI: 0.51–1.06; *P* trend = 0.05). There were no significant associations between concentrations of leptin, MCP1, and PAI1 and pancreatic cancer risk. In a sensitivity analysis where we excluded participants with <1 year of follow‐up, the association was essentially unchanged for sRAGE. There was a nonsignificant inverse association between adiponectin and pancreatic cancer risk (Table S1).

**Table 2:** Association between baseline serum levels of biomarkers and risk of incident pancreatic cancer in the WHI Study, 1993–2013 (472 cases and 802 controls)

Biomarkers (range)	Q1	Q2	Q3	Q4	*P* trend
sRAGE (pg/mL)	242–1020	1021–1333	1334–1736	1737–6999	
Case/Control (*n*/*n*)	137/200	132/201	104/200	99/201	
OR (95% CI)^a^	1.00	0.91 (0.66–1.24)	0.74 (0.53–1.03)	0.69 (0.49–0.97)	0.02
OR (95% CI)^b^	1.00	0.95 (0.69–1.30)	0.77 (0.55–1.08)	0.70 (0.50–0.99)	0.03
OR (95% CI)^c^	1.00	0.97 (0.70–1.33)	0.78 (0.56–1.10)	0.74 (0.52–1.05)	0.05
Adiponectin (*μ*g/mL)	2.1–7.8	7.9–10.8	10.9–15.4	15.5–60.6	
Case/Control (*n*/*n*)	135/200	137/201	113/196	109/205	
OR (95% CI)^a^	1.00	1.02 (0.74–1.39)	0.85 (0.61–1.18)	0.73 (0.51–1.03)	0.04
OR (95% CI)^b^	1.00	1.05 (0.76–1.46)	0.88 (0.62–1.24)	0.74 (0.51–1.06)	0.05
OR (95% CI)^c^	1.00	1.07 (0.77–1.48)	0.92 (0.65–1.30)	0.78 (0.54–1.28)	0.14
Leptin (ng/mL)	0.7–13.3	13.2–23.2	23.2–39.7	39.7–36.5	
Case/Control (*n*/*n*)	118/200	112/201	141/200	123/201	
OR (95% CI)^a^	1.00	0.94 (0.67–1.30)	1.20 (0.87–1.66)	1.03 (0.74–1.43)	0.55
OR (95% CI)^b^	1.00	0.92 (0.66–1.30)	1.19 (0.86–1.66)	1.01 (0.73–1.41)	0.61
OR (95% CI)^c^	1.00	0.90 (0.63–1.28)	1.09 (0.74–1.60)	0.77 (0.50–1.20)	0.43
Adiponectin/Leptin	208–227	227–465	465–1050	1050–2800	
Case/Control (*n*/*n*)	131/201	138/200	123/201	102/200	
OR (95% CI)^a^	1.00	1.06 (0.76–1.47)	0.93 (0.67–1.29)	0.75 (0.53–1.07)	0.09
OR (95% CI)^b^	1.00	1.12 (0.80–1.56)	0.96 (0.69–1.35)	0.77 (0.54–1.11)	0.12
OR (95% CI)^c^	1.00	1.27 (0.89–1.83)	1.12 (0.76–1.66)	0.90 (0.57–1.42)	0.55
MCP1 (pg/mL)	43.3–165	166–219	220–278	279–3043	
Case/Control (*n*/*n*)	114/200	117/201	121/200	142/201	
OR (95% CI)^a^	1.00	1.03 (0.73–1.46)	1.13 (0.80–1.60)	1.18 (0.84–1.68)	0.28
OR (95% CI)^b^	1.00	1.07 (0.76–1.52)	1.13 (0.79–1.61)	1.18 (0.83–1.69)	0.33
OR (95% CI)^c^	1.00	1.08 (0.76–1.54)	1.13 (0.76–1.54)	1.16 (0.81–1.65)	0.41
PAI1 (ng/mL)	3.0–59.4	59.4–75.6	75.6–93.7	93.7–205.7	
Case/Control (*n*/*n*)	101/200	128/201	133/200	132/201	
OR (95% CI)^a^	1.00	1.28 (0.91–1.81)	1.34 (0.94–1.91)	1.35 (0.94–1.93)	0.12
OR (95% CI)^b^	1.00	1.25 (0.88–1.77)	1.28 (0.90–1.84)	1.30 (0.91–1.87)	0.18
OR (95% CI)^c^	1.00	1.25 (0.88–1.77)	1.23 (0.86–1.77)	1.24 (0.86–1.80)	0.66

CI, confidence interval; MCP, monocyte chemotactic protein 1; OR, odds ratio; PAI1, plasminogen activator inhibitor‐1; sRAGE, soluble receptor for advanced glycation end products.

Univariate model.

Multivariate model adjusted for smoking status and treated type 2 diabetes.

Multivariate model adjusted for BMI (BMI < 25, BMI25~30 and BMI ≥30 kg/m^2^) in addition to model 2.

Table 3 shows that there was no significant effect modification by BMI for the observed associations between pancreatic cancer and sRAGE, adiponectin, leptin, or PAI1. BMI was, however, a potential effect modifier for the association between MCP1 and pancreatic cancer (*P* value for interaction = 0.03) with the positive association seen only among women with BMI <25 kg/m^2^ (aOR_Q4 vs Q1 _= 2.55, 95% CI: 1.41–4.61).

**Table 3:** Association between sRAGE, adiponectin, leptin, MCP1, PAI1, and adiponectin/leptin ratio and risk of incident pancreatic cancer by BMI status in the WHI study (472 cases and 802 controls)

Biomarkers	Cases/Controls	OR^a^	95% CI	Cases/Controls	OR^a^	95% CI
	BMI <25 kg/m^2^			BMI ≥25 kg/m^2^		
sRAGE (pg/mL)						
Q1	38/51	1.00		99/149	1.00	
Q2	47/61	0.94	0.52–1.70	85/140	0.96	0.65–1.41
Q3	34/83	0.54	0.29–0.99	70/177	0.94	0.63–1.41
Q4	49/106	0.62	0.35–1.10	50/95	0.80	0.51–1.24
*P* for trend	0.04			0.35		
*P* for interaction	0.18					
Adiponectin (*μ*g/mL)						
Q1	30/49	1.00		98/151	1.00	
Q2	48/57	1.49	0.79–2.79	84/144	0.96	0.65–1.42
Q3	38/74	1.02	0.53–1.94	72/122	0.98	0.65–1.48
Q4	52/121	0.85	0.46–1.57	50/84	0.98	0.62–1.56
*P* for trend	0.22			0.95		
*P* for interaction	0.14					
Leptin (ng/mL)						
Q1	89/159	1.00		23/41	1.00	
Q2	50/101	0.68	0.43–1.10	56/100	0.97	0.52–1.79
Q3	23/35	0.74	0.38–1.42	114/165	1.21	0.68–2.11
Q4	5/6	1.28	0.35–4.76	111/195	0.84	0.46–1.53
*P* for trend	0.38			0.55		
*P* for interaction	0.91					
Adiponectin/Leptin						
Q1	9/7	1.00		117/194	1.00	
Q2	30/41	0.56	0.18–1.75	100/159	1.21	0.83–1.75
Q3	53/95	0.53	0.18–1.58	66/106	1.14	0.75–1.75
Q4	76/158	0.50	0.17–1.50	21/42	1.02	0.55–1.89
*P* for trend	0.36			0.73		
*P* for interaction	0.12					
MCP1 (pg/mL)						
Q1	30/76	1.00		79/124	1.00	
Q2	46/83	1.33	0.75–2.36	66/118	0.89	0.58–1.37
Q3	38/85	1.06	0.58–1.93	81/115	1.06	0.70–1.61
Q4	54/57	2.55	1.41–4.61	78/144	0.79	0.52–1.19
*P* for trend	0.007			0.38		
*P* for interaction	0.03					
PAI1 (ng/mL)						
Q1	39/96	1.00		57/104	1.00	
Q2	56/89	1.37	0.82–2.30	68/112	1.06	0.68–1.67
Q3	35/65	1.22	0.64–2.06	92/135	1.20	0.79–1.84
Q4	38/51	1.53	0.85–2.74	87/150	0.94	0.60–1.45
*P* for trend	0.25			0.86		
*P* for interaction	0.32					

BMI, body mass index; CI, confidence interval; MCP, monocyte chemotactic protein 1; OR, odds ratio; PAI1, plasminogen activator inhibitor‐1; sRAGE, soluble receptor for advanced glycation end products.

Multivariate model adjusted for age, race/ethnicity, smoking status, treated type 2 diabetes, and BMI (continuous).

Table 4 shows weak‐to‐moderate correlation among all circulating biomarkers and with BMI among controls. All the biomarkers, except for MCP1, were statistically significantly correlated with BMI as well as with each other. sRAGE had significant weak negative correlation with BMI and leptin, but a positive correlation with adiponectin. Leptin was statistically significantly correlated with all the biomarkers and had the strongest positive correlation with BMI. There was a significant positive correlation between MCP1 and PAI1. Both sRAGE and adiponectin had no significant correlation with either MCP1 or PAI1. The correlation among biomarkers in the cases followed the same pattern (data not shown).

**Table 4:** Correlations of measured biomarkers and BMI among 802 controls

	log(sRAGE)	log(adiponectin)	log(leptin)	log(MCP1)	log(PAI1)	log(BMI)
log(sRAGE) (*r*)	1	0.18761	−0.17986	0.02997	−0.09204	−0.23942
*P* value		<0.0001	<0.0001	0.3472	0.0038	<0.0001
log(adiponectin) (*r*)		1	−0.34983	0.06862	−0.0592	−0.31378
*P* value			<0.0001	0.0312	0.0631	<0.0001
log(leptin) (*r*)			1	0.12813	0.21095	0.73404
*P* value				<0.0001	<0.0001	<0.0001
log(MCP1) (*r*)				1	0.30799	0.09832
*P* value					<0.0001	0.002
log(PAI1) (*r*)					1	0.22814
*P* value						<0.0001
log(BMI) (*r*)						1
*P* value						

Based on the inter‐relationship among the biomarkers, the PCA analysis identified two composite biomarkers (A and B). Composite biomarker A (anti‐inflammatory) was positively significantly correlated with sRAGE and adiponectin, negatively correlated with leptin, but not correlated with MCP1 or PAI1. Composite biomarker B (proinflammatory) was positively correlated with MCP1, PAI1, and leptin, but not correlated with sRAGE and adiponectin. These two components yielded well‐defined exploratory factors that tracked the syndemic behavior of the five biomarkers. We found, like results for the individual biomarkers, a suggestion of potential significant effect modification by BMI for composite biomarker score B, which was associated with increased risk of pancreatic cancer only in women with BMI <25 kg/m^2^ (aOR _Q4 vs. Q1 _= 1.82 per unit increase in score B, 95% CI: 1.04–3.18), but not in women with BMI ≥25 kg/m^2^. The composite biomarker A showed no association with risk of incident pancreatic cancer (Table 5).

**Table 5:** Confirmatory factor analysis based on two composite biomarkers identified by PCA and risk of incident pancreatic cancer by BMI (472 cases and 802 controls)

	OR^a^ (95% CI)
All	
Composite biomarker A^a^	0.99 (0.79–1.24)
Composite biomarker B^a^	1.26 (0.90–1.78)
BMI <25 kg/m^2^	
Composite biomarker A^a^	1.82 (1.04–3.18)
BMI ≥25 kg/m^2^	
Composite biomarker B^a^	1.03 (0.68–1.57)

The conditional logistic regression model was adjusted for smoking status and treated type 2 diabetes.

The composite biomarker includes positively loaded sRAGE, adiponectin, and negatively loaded leptin.

a The composite biomarker includes positively loaded leptin, MCP1, and PAI1.

Table S2 shows there was no statistically significant interaction between any of the biomarkers and follow‐up years on pancreatic cancer risk. Nevertheless, we observed a suggestive significant inverse association between leptin and risk of pancreatic cancer among women who were followed up for <10 years, whereas leptin was associated with a significant increased risk of pancreatic cancer among women who were followed up for ≥10 years.

In a sensitivity analysis where we included controls who later developed cancers of breast, colon–rectum, and lung during follow‐up and their matched pancreatic cancer cases, the association between sRAGE and pancreatic cancer was only moderately attenuated and became nonsignificant, while risk estimates for PAI1 were attenuated more than 10%. The observed interaction between MCP1 and BMI remained the same (Tables S3 and S4).

## Discussion

In this nested case–control study in the WHI Study, we prospectively examined the association between baseline serum concentrations of sRAGE and multiple adipokines/chemokines and the risk of incident pancreatic cancer. We found that sRAGE was inversely associated with pancreatic cancer risk in postmenopausal women regardless of BMI, smoking status or follow‐up time. We also found that lean women (BMI < 25 kg/m^2^) with higher MCP1 had increased risk of pancreatic cancer. Furthermore, lean women with a higher proinflammatory composite biomarker score comprised of MCP1, PAI1, and leptin had an increased risk of pancreatic cancer.

Our observation of an inverse association between sRAGE and pancreatic cancer agreed with our earlier finding that sRAGE was inversely associated with pancreatic cancer in older Finnish male smokers 15. The consistency is particularly notable given that these studies were conducted in geographical‐ and gender‐distinct populations, thus suggesting that sRAGE may play an important role in development of pancreatic cancer across a range of risk factors. sRAGE may neutralize a shared detrimental effect of RAGE signaling induced by major risk factors for pancreatic cancer within each cohort, that is, smoking (the Finnish male cohort) or overweight/obesity (the WHI cohort). RAGE signaling has been shown to be an important part of innate immunity and to play a role in regulating atherogenesis, angiogenic response, vascular injury, autophagy, and inflammatory response 30, 31, 32, 33, with RAGE considered as a potential antitumor therapeutic target 32.

RAGE expressed in adipocytes has been shown to modulate expression of adipokines 16. In our study, serum sRAGE was positively correlated with serum adiponectin but negatively correlated with leptin. Serum concentrations of adiponectin were nonsignificantly inversely associated with pancreatic cancer risk, with the association more pronounced when excluding study participants followed up for <1 year. This result was largely consistent with several previous prospective studies 19, 22, 24, as were our findings on no association between leptin concentrations and overall risk of pancreatic cancer 18. However, we found that higher leptin concentrations were associated with increased risk of pancreatic cancer in those who were followed up for ≥10 years, but with significantly decreased risk in those who were followed up <10 years. A pooled analysis of large prospective studies also reported a potential effect modification of the association with leptin by follow‐up time 23. We speculated that the shorter duration of follow‐up may be related to precancer diagnosis‐associated weight loss as a small loss of weight could result in a reduction in leptin concentrations.

Although sRAGE, adiponectin, and leptin were correlated with BMI, we did not observe statistically significant effect modification of the associations for these biomarkers by BMI. Nevertheless, we found the significant effect modification of MCP1 by BMI, with an increased risk only seen in women with normal BMI. Effect modification by BMI of the association between pancreatic cancer risk and the proinflammatory composite biomarker comprising MCP1, PAI1, and leptin was also seen.

The association between circulating MCP1 and PA1 and risk of pancreatic cancer has not been examined previously. CC chemokines, especially MCP1 (CCL2) and CCL5, are major attractants of monocyte and macrophage precursors to the tumor microenvironment. Pancreatic cancer uses the CCL2/CCL2 receptor (CCR2) axis to favor the mobilization and recruitment of inflammatory monocytes from the bone marrow to the tumor site to facilitate tumor growth 25, 34. MCP1 also potently activates urokinase plasminogen activator (uPA). uPA is an enzyme responsible for cleavage of plasminogen to plasmin which mediates the degradation of the extracellular matrix for cancer cell invasion and has been shown to contribute to the invasive behavior of pancreatic cancer 35. PAI1's main function entails the inhibition of uPA and thus inhibits the activity of tissue plasminogen activator and fibrinolysis 36. Paradoxically, protumorigenic and proangiogenic properties of PAI1 have been established 26, which are in line with our finding. Leptin has been shown to stimulate the synthesis of inflammatory mediators such as MCP1 and ICAM1 in lean rats 37 and to upregulate the expression of PAI1 in endothelial cells via the activation of ERK1/2 38. Concordantly, we found that a higher score of proinflammatory composite biomarker comprising MCP1, PAI1, and leptin was associated with an increased risk of pancreatic cancer. Our present study indicated that MCP1 contributes to inflamed tumor microenvironment and the increase of chemokines can be detected in prediagnostic blood in lean women, and it may operate with PAI1 and leptin as shown by the composite score analysis. Although AGEs have been shown to upregulate MCP1 expression in human mesangial cells 39, and circulating AGEs and sRAGE levels have been shown to be the independent determinants of MCP1 levels in patients with type 2 diabetes 40, the correlation and composite score analyses did not support the cooperation between RAGE and MCP1 in our study population.

Because inflamed adipose tissue in obese persons has been associated with various cancers, we originally hypothesized that proinflammatory adipokines/chemokines would be associated with increased risk of pancreatic cancer particularly in obese women. However, we observed a positive association between prediagnostic concentrations of MCP1 and pancreatic cancer only in lean women. It is well known that MCP1 is expressed by a wide range of cells including endothelial, epithelial, myeloid and smooth muscle cells, and fibroblasts, either constitutively or after induction, and is a potent chemoattractant for monocytes, basophils, T lymphocytes, and NK cells 41. MCP1 is also expressed in human pancreatic cancer 42. We thus speculated that the higher concentrations of MCP1 in lean women who subsequently develop pancreatic cancer may indicate an early heightened inflammatory response from macrophages, tumor cells, endothelial cells, or other cells in the tumor microenvironment. Monti et al. 42 found that patient with high circulating levels of MCP1 had significantly higher survival rate than low MCP1 producers. The biological consequence of tumor macrophage infiltration induced by CCL2 in pancreatic cancer may be differed by adiposity. This hypothesis needs to be tested in future experimental studies. Additional larger cohort‐based research is needed to investigate potential complex interactions among multiple risk factors (e.g., biomarker‐adiposity‐risk factor‐dietary intake) that may help explain observed differences in findings in lean versus obese women.

Our study has novelty and multiple strengths. First, this study was the first to evaluate sRAGE as a risk marker for pancreatic cancer in women and to evaluate chemokines MCP1 and PAI‐1 and pancreatic cancer risk in any cohort. Second, the use of PCA to generate composite biomarker score which was interrogated in exploratory multivariable analysis was novel. Third, it was performed in the large, well‐phenotyped, and prospectively followed WHI cohort. Collection of baseline samples in women followed for an average of 14 years helped reduce concern about potential reverse causation bias, with results from our lag analysis suggesting that only the biomarker leptin may be differentially associated with disease risk over time. Finally, we evaluated multiple established and potential confounders. Methodologically, we found risk set sampling of controls in the nested case–control study may attenuate the association if matched controls had undiagnosed cancers because the biomarker abnormality may have occurred in undiagnosed controls.

There are several limitations of our study. First, the generalizability of our findings on MCP1 is limited because the postmenopausal women in our study were largely non‐Hispanic White and never or former smokers. In addition, given the tumor microenvironment may be organ‐specific, these study findings may not be generalizable to other types of cancer. Second, given the rarity of pancreatic cancer, study power was limited in stratified analyses. Our results were not adjusted for multiple testing in considering sRAGE was the major biomarker of interest. Finally, our findings on the composite biomarker suggesting excess risk in lean women only were qualified as suggestive and provisional until replicated in other cohorts.

In summary, together with our previous study 15, we showed higher serum concentrations of the anti‐inflammatory sRAGE were robustly associated with reduced risk of developing pancreatic cancer. Our present study also suggested higher concentrations of the chemokine MCP1 were associated with excess risk of pancreatic cancer in lean women. Our study advanced knowledge on dysregulation of adipokines in pancreatic cancer in the context of the sRAGE which showed that sRAGE‐adiponectin and MCP‐PAI1 act differently in pancreatic cancer carcinogenesis, while leptin is involved in both RAGE‐ and MCP1‐related mechanisms. Additional experimental and epidemiological research evaluating the potential use of sRAGE and adipokines/chemokines as preventative or therapeutic targets, or in risk stratification is warranted.

## Novelty & Impact Statements

RAGE expressed on adipocytes may modulate adipokine production. Serum concentrations of anti‐inflammatory soluble RAGE were inversely associated with risk of incident pancreatic cancer in postmenopausal women. Chemokine MCP1 and a proinflammatory biomarker score including MCP1, leptin, and PAI1 were associated with increased risk of incident pancreatic cancer among lean women. However, sRAGE was not correlated with MCP1. This research provides novel insight into obesity‐related pancreatic cancer in the context of RAGE signaling pathway and adipokine/chemokines.

## Conflict of Interest

None delared.

## Supporting information


**Table S1.** Association between baseline serum levels of biomarkers and risk of incident pancreatic cancer in the WHI Study, follow up no less than 1 year, 1993–2013 (450 cases and 801 controls).
**Table S2.** Association between sRAGE, adiponectin, leptin, MCP1 And PAI 1 and risk of pancreatic cancer by follow‐up years (472 cases and 802 controls).
**Table S3.** Association between baseline serum levels of biomarkers and risk of incident pancreatic cancer from the WHI Study, 1993–2013 (494 cases and 986 controls).
**Table S4.** Association between MCP1 and risk of pancreatic cancer by BMI status (494 cases and 986 controls).
