# Supplementary material for: A prospective study of soluble receptor for advanced glycation end products and adipokines in association with pancreatic cancer in postmenopausal women
Source: Cancer Med. 2018 Mar 23;7(5):2180–91. doi: 10.1002/cam4.1426 (PMC5943487; doi:10.1002/cam4.1426)
Supplement: Supplementary file 1 — Table S1. Association between baseline serum levels of biomarkers and risk of incident pancreatic cancer in the WHI Study, follow up no less than 1 year, 1993–2013 (450 cases and 801 controls). Table S2. Association between sRAGE, adiponectin, leptin, MCP1 And PAI 1 and risk of pancreatic cancer by follow‐up years (472 cases and 802 controls). Table S3. Association between baseline serum levels of biomarkers and risk of incident pancreatic cancer from the WHI Study, 1993–2013 (494 cases and 986 controls). Table S4. Association between MCP1 and risk of pancreatic cancer by BMI status (494 cases and 986 controls). [file CAM4-7-2180-s001.docx]

**Supplemental table 1. Association between baseline serum levels of biomarkers and risk of incident pancreatic cancer in the WHI Study, follow up no less than 1 year, 1993-2013 (450 cases and 801 controls)**

| **Biomarkers (range)** | **Q1** | **Q2** | **Q3** | **Q4** | | ***P* _trend_** | |  | |
| --- | --- | --- | --- | --- | --- | --- | --- | --- | --- |
| **sRAGE (pg/ml)** | (242-1020) | (1021-1333) | (1334-1736) | | (1737-6999) | |  | |  |
| Case/Control (n/n) | 130/199 |  |  | |  | |  | |  |
| OR (95% CI)^a^ | 1.00 | 0.94 (0.68-1.30) | 0.80 (0.57-1.13) | | 0.70 (0.49-1.01) | | 0.04 | |  |
| OR (95% CI)^b^ | 1.00 | 0.96 (0.69-1.34) | 0.82 (0.58-1.17) | | 0.74 (0.52-1.07) | | 0.08 | |  |
|  |  |  |  | |  | |  | |  |
| **Adiponectin (µg/ml)** | (2.1-7.8) | (7.9-10.8) | (10.9-15.4) | | (15.5-60.6) | |  | |  |
| Case/Control (n/n) | 123/199 | 126/201 | 105/196 | | 96/205 | |  | |  |
| OR (95% CI)^a^ | 1.00 | 1.00 (0.72-1.40) | 0.84 (0.59-1.19) | | 0.70 (0.48-1.01) | | 0.03 | |  |
| OR (95% CI)^b^ | 1.00 | 1.02 (0.73-1.43) | 0.88 (0.62-1.26) | | 0.74 (0.51-1.09) | | 0.09 | |  |

|  |  |  |  |  |  |  |
| --- | --- | --- | --- | --- | --- | --- |
| **Leptin (ng/ml)** | (0.7-13.3) | (13.2-23.2) | (23.2-39.7) | (39.7-36.5) |  |  |
| Case/Control (n/n) | 102/200 | 101/201 | 135/199 | 112/201 |  |  |
| OR (95% CI)^a^ | 1.00 | 0.98 (0.69-1.39) | 1.29 (0.92-1.81) | 1.06 (0.76-1.50) | 0.43 |  |
| OR (95% CI)^b^ | 1.00 | 0.95 (0.66-1.37) | 1.18 (0.79-1.75) | 0.80 (0.51-1.26) | 0.56 |  |
|  |  |  |  |  |  |  |
| **Adiponectin/Leptin** | (208-227) | (227-465) | (465-1050) | (1050-2800) |  |  |
| Case/Control (n/n) | 121/200 | 130/200 | 108/201 | 91/200 |  |  |
| OR (95% CI)^a^ | 1.00 | 1.16 (0.83-1.62) | 0.89 (0.63-1.26) | 0.75 (0.52-1.09) | 0.07 |  |
| OR (95% CI)^b^ | 1.00 | 1.33 (0.92-1.92) | 1.04 (0.70-1.55) | 0.88 (0.55-1.41) | 0.41 |  |
|  |  |  |  |  |  |  |
| **MCP1 (pg/ml)** | (43.3-165) | (166-219) | (220-278) | (279-3043) |  |  |
| Case/Control (n/n) | 105/200 | 106/201 | 116/199 | 123/201 |  |  |
| OR (95% CI)^a^ | 1.00 | 1.03 (0.72-1.48) | 1.20 (0.83-1.72) | 1.14 (0.80-1.64) | 0.35 |  |
| OR (95% CI)^a^ | 1.00 | 1.05 (0.73-1.50) | 1.21 (0.84-1.74) | 1.12 (0.78-1.61) | 0.43 |  |
|  |  |  |  |  |  |  |
| **PAI1 (ng/ml)** | (3.0-59.4) | (59.4-75.6) | (75.6-93.7) | (93.7-205.7) |  |  |
| Case/Control (n/n) | 94/200 | 120/200 | 121/200 | 115/201 |  |  |
| OR (95% CI)^a^ | 1.00 | 1.21 (0.85-1.72) | 1.22 (0.85-1.76) | 1.26 (0.86-1.82) | 0.27 |  |
| OR (95% CI)^b^ | 1.00 | 1.21 (0.85-1.72) | 1.16 (0.80-1.72) | 1.18 (0.80-1.73) | 0.49 |  |
|  |  |  |  |  |  |  |

^a^ Multivariate model adjusted for smoking status and treated type 2 diabetes.

^b^ Multivariate model adjusted for smoking status, diabetes and BMI(BMI<25, BMI25~30 and BMI≥30 kg/m^2^).

**Supplemental table 2. Association between sRAGE, adiponectin, leptin, MCP1 And PAI 1 and risk of pancreatic cancer by follow-up years (472 cases and 802 controls)**

|  | Follow up<10 yrs | OR^a^ | (95% CI) | Follow up≥10 yrs | OR^a^ | (95% CI) |
| --- | --- | --- | --- | --- | --- | --- |
| sRAGE (pg/ml) |  |  |  |  |  |  |
| Q1 | 72/18 | 1.00 |  | 65/182 | 1.00 |  |
| Q2 | 67/24 | 0.72 | (0.35-1.48) | 65/177 | 1.01 | (0.67-1.52) |
| Q3 | 55/24 | 0.60 | (0.29-1.24) | 49/176 | 0.74 | (0.48-1.15) |
| Q4 | 42/24 | 0.46 | (0.22-0.98) | 57/177 | 0.82 | (0.53-1.28) |
| *P* for trend | 0.04 |  |  | 0.21 |  |  |
| *P* for interaction | 0.32 |  |  |  |  |  |
|  |  |  |  |  |  |  |
| Adiponectin (µg/ml) |  |  |  |  |  |  |
| Q1 | 68/26 | 1.00 |  | 60/174 | 1.00 |  |
| Q2 | 63/28 | 0.87 | (0.44-1.71) | 69/173 | 1.15 | (0.75-1.75) |
| Q3 | 51/17 | 1.10 | (0.51-2.38) | 59/179 | 0.96 | (0.62-1.50) |
| Q4 | 54/19 | 1.16 | (0.54-2.50) | 48/186 | 0.73 | (0.46-1.18) |
| *P* for trend | 0.57 |  |  | 0.14 |  |  |
| *P* for interaction | 0.16 |  |  |  |  |  |
|  |  |  |  |  |  |  |
| Leptin (ng/ml) |  |  |  |  |  |  |
| Q1 | 63/14 | 1.00 |  | 49/186 | 1.00 |  |
| Q2 | 49/22 | 0.43 | (0.19-0.97) | 57/179 | 1.24 | (0.79-1.96) |
| Q3 | 58/26 | 0.37 | (0.15-0.90) | 79/174 | 1.79 | (1.10-2.90) |
| Q4 | 66/28 | 0.26 | (0.10-0.70) | 51/173 | 1.12 | (0.63-2.02) |
| *P* for trend | 0.01 |  |  | 0.35 |  |  |
| *P* for interaction | 0.25 |  |  |  |  |  |
|  |  |  |  |  |  |  |
| MCP1 (pg/ml) |  |  |  |  |  |  |
| Q1 | 61/20 | 1.00 |  |  | 1.00 |  |
| Q2 | 45/21 | 0.76 | (0.34-1.63) |  | 1.40 | (0.91-2.16) |
| Q3 | 62/21 | 1.00 | (0.48-2.10) |  | 1.17 | (0.75-1.84) |
| Q4 | 68/28 | 0.84 | (0.41-1.71) |  | 1.43 | (0.91-2.22) |
| *P* for trend | 0.81 |  |  |  | 0.23 |  |
| *P* for interaction | 0.75 |  |  |  |  |  |
|  |  |  |  |  |  |  |
| PAI1 (ng/ml) |  |  |  |  |  |  |
| Q1 | 51/24 | 1.00 |  |  | 1.00 |  |
| Q2 | 57/27 | 0.94 | 0.48-1.88 |  | 1.46 | 0.94-2.26 |
| Q3 | 60/18 | 1.52 | 0.72-3.21 |  | 1.40 | 0.90-2.18 |
| Q4 | 68/21 | 1.43 | 0.68-3.00 |  | 1.21 | 0.76-1.92 |
| *P* for trend | 0.19 |  |  |  | 0.52 |  |
| *P* for interaction | 0.69 |  |  |  |  |  |

^a^ Multivariate model adjusted for age, ethinicity, BMI(BMI<25, BMI25~30 and BMI≥30 kg/m^2^), smoking status, and diabetes.

**Supplemental table 3. Association between baseline serum levels of biomarkers and risk of incident pancreatic cancer from the WHI Study, 1993-2013 (494 cases and 986 controls)**

| **Biomarkers (range)** | **Q1** | **Q2** | **Q3** | **Q4** | ***P* _trend_** |
| --- | --- | --- | --- | --- | --- |
| **sRAGE (pg/ml)** | (242-1016) | (1017-1344) | (1345-1737) | (1738-6999) |  |
| Case/Control (n/n) | 138/246 | 149/247 | 103/246 | 104/247 |  |
| OR (95% CI)^a^ | 1.00 | 1.06 (0.79-1.42) | 0.74 (0.54-1.02) | 0.73 (0.53-1.01) | 0.01 |
| OR (95% CI)^b^ | 1.00 | 1.07 (0.79-1.44) | 0.75 (0.54-1.02) | 0.74 (0.54-1.03) | 0.02 |
| OR (95% CI)^c^ | 1.00 | 1.08 (0.80-1.46) | 0.75 (0.55-1.04) | 0.75 (0.54-1.04) | 0.02 |
|  |  |  |  |  |  |
| **Adiponectin (µg/ml)** | (2.1-7.8) | (7.9-10.8) | (10.9-15.4) | (15.5-60.6) |  |
| Case/Control (n/n) | 132/242 | 132/243 | 121/249 | 109/252 |  |
| OR (95% CI)^a^ | 1.00 | 0.98 (0.72-1.33) | 0.87 (0.64-1.19) | 0.77 (0.55-1.07) | 0.09 |
| OR (95% CI)^b^ | 1.00 | 1.00 (0.73-1.37) | 0.91 (0.66-1.26) | 0.79 (0.56-1.12) | 0.15 |
| OR (95% CI)^c^ | 1.00 | 1.01 (0.74-1.38) | 0.93 (0.67-1.29) | 0.82 (0.57-1.16) | 0.23 |

|  |  |  |  |  |  |
| --- | --- | --- | --- | --- | --- |
| **Leptin (ng/ml)** | (0.7-13.2) | (13.2-23.2) | (23.2-39.7) | (39.7-365.3) |  |
| Case/Control (n/n) | 123/246 | 117/247 | 144/246 | 110/247 |  |
| OR (95% CI)^a^ | 1.00 | 0.96 (0.71-1.31) | 1.18 (0.87-1.60) | 0.89 (0.65-1.23) | 0.81 |
| OR (95% CI)^b^ | 1.00 | 0.95 (0.70-1.31) | 1.18 (0.87-1.61) | 0.89 (0.64-1.23) | 0.81 |
| OR (95% CI)^c^ | 1.00 | 0.93 (0.66-1.30) | 1.10 (0.76-1.58) | 0.70 (0.46-1.08) | 0.24 |
|  |  |  |  |  |  |
| **Adiponectin/Leptin** | (208-227) | (227-465) | (465-1050) | (1050-2800) |  |
| Case/Control (n/n) | 126/247 | 133/246 | 126/246 | 109/247 |  |
| OR (95% CI)^a^ | 1.00 | 1.06 (0.78-1.45) | 1.00 (0.73-1.36) | 0.86 (0.62-1.19) | 0.33 |
| OR (95% CI)^b^ | 1.00 | 1.13 (0.82-1.56) | 1.04 (0.76-1.43) | 0.92 (0.65-1.27) | 0.55 |
| OR (95% CI)^c^ | 1.00 | 1.22 (0.86-1.73) | 1.13 (0.78-1.63) | 0.98 (0.63-1.51) | 0.82 |
|  |  |  |  |  |  |
| **MCP-1 (pg/ml)** | (43.3-165) | (166-219) | (220-278) | (279-3043) |  |
| Case/Control (n/n) | 117/245 | 116/247 | 126/247 | 135/247 |  |
| OR (95% CI)^a^ | 1.00 | 1.00 (0.72-1.37) | 1.09 (0.79-1.50) | 1.18 (0.85-1.65) | 0.27 |
| OR (95% CI)^b^ | 1.00 | 1.03 (0.74-1.42) | 1.10 (0.79-1.53) | 1.18 (0.84-1.65) | 0.30 |
| OR (95% CI)^c^ | 1.00 | 1.03 (0.74-1.44) | 1.10 (0.79-1.53) | 1.16 (0.83-1.63) | 0.35 |
|  |  |  |  |  |  |
| **PAI-1 (ng/ml)** | (3.0-59.4) | (59.4-75.6) | (75.6-93.7) | (93.7-205.7) |  |
| Case/Control (n/n) | 108/246 | 130/247 | 129/246 | 127/247 |  |
| OR (95% CI)^a^ | 1.00 | 1.22 (0.89-1.67) | 1.22 (0.88-1.70) | 1.21 (0.86-1.69) | 0.32 |
| OR (95% CI)^b^ | 1.00 | 1.18 (0.85-1.63) | 1.16 (0.83-1.62) | 1.16 (0.83-1.64) | 0.45 |
| OR (95% CI)^c^ | 1.00 | 1.18 (0.85-1.63) | 1.14 (0.82-1.59) | 1.13 (0.80-1.60) | 0.58 |

^a^ Univariate model

^b^ Multivariate model adjusted for smoking and diabetes.

^c^ Multivariate model adjusted for BMI(BMI<25, BMI25~30 and BMI≥30 kg/m^2^) in addition to model 2.

**Supplemental table 4. Association between MCP1 and risk of pancreatic cancer by BMI status (494 cases and 986 controls)**

| **Factors** | **Cases/**  **Control** | **OR^a^** | | **(95% CI)** | | **Cases/**  **Controls** | **OR^a^** | **(95% CI)** |
| --- | --- | --- | --- | --- | --- | --- | --- | --- |
|  |  |  |  | |  | |  |  |
| **MCP1 (pg/ml)** |  |  |  | |  | |  |  |
|  | **BMI <25 kg/m^2^** | |  | | **BMI ≥ 25 kg/m^2^** | | |  |
| Q1 | 33/93 | 1.00 |  | | 84/152 | | 1.00 |  |
| Q2 | 48/103 | 1.25 | (0.73-2.15) | | 68/144 | | 0.86 | (0.57-1.29) |
| Q3 | 42/99 | 1.14 | (0.65-2.00) | | 84/148 | | 1.01 | (0.68-1.50) |
| Q4 | 52/69 | 2.19 | (1.25-3.85) | | 83/178 | | 0.80 | (0.54-1.18) |
| *P* for trend | 0.008 |  |  | | 0.39 | |  |  |
| *P* for interaction | 0.04 |  |  | |  | |  |  |

^a^ Multivariate model adjusted for age, ethinicity, smoking status, treated type 2 diabetes and BMI (continuous).
